# Balance and Mobility in Comparison to Patient-Reported Outcomes—A Longitudinal Evaluation After Total Hip and Knee Arthroplasty

**DOI:** 10.3390/jcm14124135

**Published:** 2025-06-11

**Authors:** Klemens Vertesich, Kevin Staats, Eleonora Schneider, Madeleine Willegger, Reinhard Windhager, Christoph Böhler

**Affiliations:** Department of Orthopedics and Trauma Surgery, Medical University of Vienna, Waehringer Guertel 18-20, 1090 Vienna, Austria; kevin.staats@meduniwien.ac.at (K.S.); eleonora.schneider@meduniwien.ac.at (E.S.); madeleine.willegger@meduniwien.ac.at (M.W.); reinhard.windhager@meduniwien.ac.at (R.W.); christoph.boehler@meduniwien.ac.at (C.B.)

**Keywords:** total hip arthroplasty, total knee arthroplasty, balance, Tinetti performance-oriented mobility assessment, mobility, patient-reported outcome measures, recovery

## Abstract

**Background:** Balance and gait are critical for functional recovery and fall prevention following total hip (THA) and knee arthroplasty (TKA). Despite improvements in pain and joint function, residual impairments often persist. The Timed Up and Go (TUG) test and Tinetti Performance-Oriented Mobility Assessment (POMA) objectively measure postoperative mobility and balance, while patient-reported outcome measures (PROMs) assess perceived function and well-being. This study longitudinally evaluates functional measurement and PROMs to explore their interrelationships and compare recovery trajectories in THA and TKA cohorts. **Methods:** In this prospective study, 22 THA and 21 TKA patients were assessed preoperatively and at 4–6 days, 6 weeks, 3 months, and 12 months postoperatively using TUG, Tinetti, Hip Disability and Osteoarthritis Outcome Score (HOOS), Knee Injury and Osteoarthritis Outcome Score (KOOS), and clinical scores (Harris Hip Score (HHS) for THA, Knee Society Score (KSS) for TKA). Pearson correlation assessed relationships between measures. **Results:** Both cohorts demonstrated significant immediate postoperative declines in balance and mobility, recovering to baseline by 6 weeks and surpassing it by 3 and 12 months (*p* < 0.001). PROMs showed earlier and sustained improvements. Objective balance and mobility measures showed minimal correlation with PROMs but were highly interrelated from 6 weeks onward. **Conclusions:** THA and TKA patients experience early postoperative balance impairments, suggesting heightened fall risk, with functional recovery lagging behind perceived well-being, highlighting the need for cautious rehabilitation strategies.

## 1. Introduction

Balance and gait are critical factors influencing the success of total hip (THA) and total knee arthroplasty (TKA), as these procedures not only aim to alleviate pain and restore joint function but also to improve overall mobility, quality of life, and patient satisfaction [1,2,3,4].

Following THA or TKA, patients generally experience significant improvements in pain levels and joint function, which in turn promote better gait patterns and balance [5,6]. Improvements in operation [7] and alignment techniques [8], cementation technique [9], implant design [10], and rehabilitation [11] lead to improved results. Despite these improvements, gait deviations and balance impairments may persist in the individual patient due to residual muscle weakness, altered proprioception, or the adaptation to a new joint alignment [12,13]. Rehabilitation programs typically include strength training, proprioceptive exercises, and gait retraining to optimize functional recovery, leading to improvements in walking speed, stride length, and overall stability, which are crucial for reducing the risk of falls and enhancing independence in daily activities [14,15,16].

The Timed Up and Go (TUG) test is a widely used clinical tool for assessing mobility in patients who have undergone THA or TKA [17,18]. The TUG test involves timing a patient as they rise from a chair, walk a short distance, turn around, walk back, and sit down. Its simplicity, minimal equipment requirements, and ease of administration make it a popular choice in both clinical and research settings. By providing a quantifiable measure of functional ambulation, the TUG test is instrumental in evaluating the effectiveness of rehabilitation programs and monitoring recovery progress over time [19,20].

The Tinetti Performance-Oriented Mobility Assessment (POMA) commonly referred to as the Tinetti score, is another established tool used to evaluate balance and gait [21,22]. The Tinetti score assesses a patient’s ability to perform tasks related to balance and gait through a series of maneuvers, such as sitting, standing, and walking. Scores on the Tinetti assessment can help clinicians identify patients at higher risk of falls, especially in elderly or neurologically and cognitive impaired populations [23,24,25,26,27].

In addition to objective performance tests, patient-reported outcome measurements (PROMs) such as the Hip Disability and Osteoarthritis Outcome Score (HOOS) and the Knee Injury and Osteoarthritis Outcome Score (KOOS) are indispensable for monitoring postoperative well-being [28,29]. These instruments capture patients’ perspectives on pain, function, and overall quality of life, offering a comprehensive view on the potential subjective impact of the surgery, that affects patients in their daily routine [30]. HOOS and KOOS assess various dimensions including daily living activities, sports and recreational function, and joint-specific symptoms, thereby guiding clinicians in tailoring rehabilitation programs to meet individual needs. The integration of PROMs into clinical practice has been associated with improved patient satisfaction and enhanced long-term outcomes, as these measures facilitate early detection of potential issues [31,32,33]. PROMs, in combination with the TUG test and other clinical assessments, are commonly utilized as monitoring tools following TKA and THA, as demonstrated in multicenter studies [11]. However, the integration of the Tinetti Score alongside TUG and PROMs has been described only to a limited extent and may offer valuable additional insights in the context of rehabilitation research following joint arthroplasty.

This study aims to evaluate functional status in patients undergoing THA or TKA using objective clinical assessments, including the Tinetti score and the TUG, as well as PROMs such as the HOOS and the KOOS, both preoperatively and postoperatively. It aims to investigate correlations between gait and balance parameters, Tinetti score and TUG test, and PROMs, in order to identify potential surrogate markers associated with an increased risk of falls in this patient population. Furthermore, it aims to compare recovery trajectories and functional outcomes between THA and TKA patients, with a focus on differences in mobility, balance, and self-reported function over time.

## 2. Methods

This study was approved on 16 January 2021 by the ethics committee of the Medical University of Vienna (Nr. 2470/2020). It was designed as a prospective observational study. The Strengthening the Reporting of Observational Studies in Epidemiology (STROBE) was used as a guideline for reporting the study [34].

### 2.1. Patients

Participants were eligible if they were between 45 and 85 years of age and scheduled for THA or TKA due to primary or secondary osteoarthritis of the affected joint. Informed consent was required, as evidenced by a signed consent form, and all subjects had to meet the necessary baseline criteria for performing the functional and balance tests; the presence of preexisting joint endoprostheses or other medical devices did not serve as exclusion criteria. Conversely, individuals with severe neuromuscular disorders, such as Parkinson’s disease or multiple sclerosis, were excluded. Additionally, patients with significant comorbidities that severely limited daily activities and would prevent them from completing the prescribed tests could not participate.

Sample size calculation was performed where published TUG data from patients following THA or TKA served as the reference. Assuming a mean improvement of 7 s in the TUG, a power analysis with 80% power and an alpha level of 0.05 indicated a required sample size of 18 participants. To accommodate the evaluation of multiple clinical and subjective parameters, a target sample size of 20 patients was set.

In total, 22 patients in the THA group and 21 patients in the TKA group were recruited for this study. In the THA group, there were 13 women (59.1%) and 9 men (40.9%). Among the TKA patients, there were 17 women (81.0%) and 4 men (19.0%). The mean age at the time of surgery for the entire cohort was 64.28 years, with a mean age of 63.98 (Standard deviation (SD) 9.60) in the THA group and 64.58 years (SD 9.37) in the TKA group.

### 2.2. Surgical Procedures and Rehabilitation Process

All total hip arthroplasty procedures were performed using a minimally invasive direct anterior approach. Acetabular reconstruction was achieved with a hemispherical modular cup (Pinnacle cup, Depuy Synthes, Raynham, MA, USA), while femoral reconstruction utilized a cementless, collared, variable triple taper stem (Actis, Depuy Synthes, Raynham, MA, USA); bearing options consisted of either ceramic-on-polyethylene or ceramic-on-ceramic articulations. Total knee arthroplasty was executed via a medial parapatellar approach using a cemented primary knee replacement system (Attune Knee System, Depuy Synthes, Raynham, MA, USA) with a posterior cruciate ligament-retaining design. Postoperatively, both cohorts initiated full weight-bearing mobilization on day one using two crutches under the supervision of physical therapists, were discharged no earlier than postoperative day six, and were advised to use crutches for six weeks. Following discharge, patients participated in a standardized active rehabilitation protocol and were routinely referred to a three-week inpatient rehabilitation program, typically commencing 8 to 12 weeks after surgery.

### 2.3. Clinical Assessments and Questionnaire

Patients underwent a standardized evaluation protocol with assessments and questionnaires administered preoperatively, on postoperative days 4–6 prior to discharge, and at 6 weeks, 3 months, and 12 months postoperatively. Demographic data were collected from all patients at the time of study enrollment, alongside an assessment of subjective well-being. The evaluation protocol began with the completion of standardized PROMs, specifically the HOOS for patients undergoing THA and the KOOS for those undergoing TKA [28,29]. Following this, a clinical examination was conducted to assess joint ROM. Subsequently, functional mobility and balance were evaluated using both the TUG test and the Tinetti score. The Timed Up and Go (TUG) test is a clinically validated tool used to assess functional mobility and was therefore chosen for this study. For the TUG test, patients began seated in a standard armchair and, upon instruction, stood up and walked a clearly marked distance of 3 m, then turned around, walked back to the chair, and sat down, with the total time recorded in seconds. The use of armrests was allowed if necessary, although their use was minimized to ensure an accurate assessment of mobility, and the test was typically repeated to obtain an average or best performance for analysis [17].

The Tinetti Score is a validated, reliable tool that measures gait and balance performance to predict fall risk and track functional mobility changes in clinical populations. It consisted of two parts: the balance component, which involved assessing tasks such as sitting stability, the ability to rise from a seated position, maintaining an upright posture during standing, and turning maneuvers; and the gait component, during which the patient’s step length, symmetry, continuity, and deviation from a straight path were observed and scored [22].

In addition to these performance-based assessments, clinical functional scores were recorded, with the Knee Society Score (KSS) applied to patients undergoing total knee arthroplasty and the Harris Hip Score (HHS) used for those receiving total hip arthroplasty. KSS resulted of two separate scales for pain (KSSp) and function (KSSf). All assessments and clinical testing were performed by the same three observers throughout the entire study. Before the enrollment of patients, observers performed instructed training assessments on 15 THA and 15 TKA patients to standardize the procedure and reduce potential investigation biases.

### 2.4. Statistical Analysis

The collected results were tested for normal distribution using the Kolmogorov–Smirnov method. Datasets that followed a normal distribution were analyzed longitudinally with paired t-tests, whereas non-normally distributed data were evaluated using the non-parametric Wilcoxon signed-rank test. Furthermore, Pearson’s correlation coefficient was used to assess the correlation between the measurements. Correlations were classified as weak (r = 0 to 0.4), moderate (r = 0.41 to 0.7), high (r = 0.71 to 0.95), and very high (r = 0.96 to 1). Due to multiple measurements, functional tests were correlated with PROMS on each individual timepoint. Independent t-tests were performed to compare the results between the two study groups (THA vs. TKA), and if normality is not assumed, the Mann–Whitney U test was used to determine statistical significance. Descriptive statistics were used to assess mean values and standard deviation (SD) of assessment results. Statistical tests were considered significant with *p*-values of less than 0.05. All statistical assessments were performed using SPSS 29.0 (IBM Co., Armonk, NY, USA) and GraphPad Prism v10 (GraphPad Software, Inc., Boston, MA, USA).

## 3. Results

### 3.1. Functional Assessments and Patient-Reported Outcomes Following Total Hip Arthroplasty

**Figure 1:**
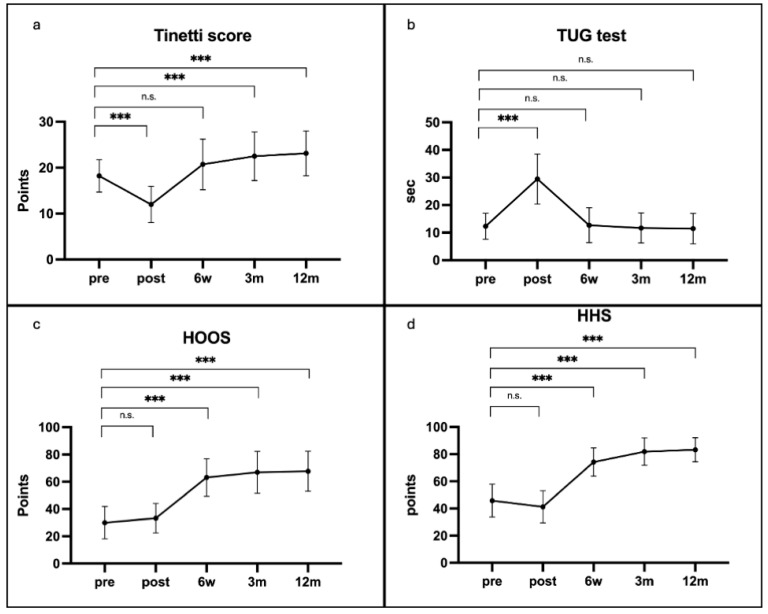
(**a**–**d**): Longitudinal trajectories of functional performance and patient-reported outcomes (PROMs) in the total hip arthroplasty (THA) cohort. Panels show mean and standard deviation scores for the Tinetti Performance-Oriented Mobility Assessment (POMA), the Timed Up and Go (TUG) test, the Hip Disability and Osteoarthritis Outcome Score (HOOS), and the Harris Hip Score (HHS) at baseline (pre), postoperative days 4–6 (post), six weeks (6 w), three months (3 m), and twelve months (12 m). Statistical comparisons to baseline are indicated as non-significant (n.s.), and *** *p* < 0.001.

Balance, as measured by the Tinetti score, exhibited a significant immediate postoperative decline (mean 18.2 (SD 3.50) preoperatively vs. 12.0 (SD 3.94) on days 4–6; *p* < 0.001), but returned to baseline by six weeks and surpassed it at three (mean 22.6 (SD 5.38)) and twelve months (mean 23.1 (SD 4.89)) (both *p* < 0.001). Functional mobility measured by the TUG test similarly worsened immediately after surgery (mean 12.3 s (SD 4.70) vs. 29.5 s (SD 9.08); *p* < 0.001) before recovering to preoperative performance by six weeks and remaining stable through one year. Table 1 presents key findings on functional assessment after THA. Figure 1a–d summarizes the key functional and patient-reported outcomes following THA.

Patient-reported HOOS and clinical hip function score HHS showed no significant change in the first postoperative week but demonstrated marked, statistically significant improvements from six weeks onward, achieving clinically meaningful gains by three months that persisted at twelve months (Figure 1a–d).

### 3.2. Functional Assessments and Patient-Reported Outcomes Following Total Knee Arthroplasty

**Figure 2:**
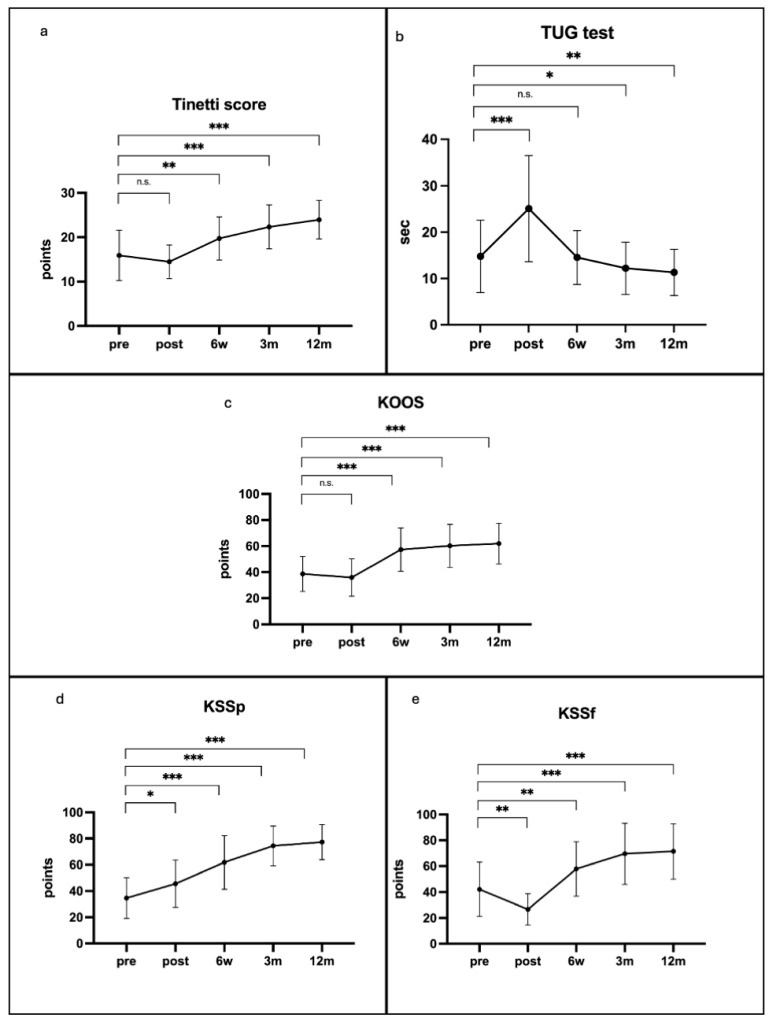
(**a**–**e**): Longitudinal trajectories of functional performance and patient-reported outcomes (PROMs) in the total knee arthroplasty (TKA) cohort. Panels show mean and standard deviation scores for the Tinetti Performance-Oriented Mobility Assessment (POMA), the Timed Up and Go (TUG) test, the Knee Injury and Osteoarthritis Outcome Score (KOOS), and the Knee Society Score (with separate pain (KSSp) and function (KSSf) subscales), at baseline (pre), postoperative days 4–6 (post), six weeks (6 w), three months (3 m), and twelve months (12 m). Statistical comparisons to baseline are indicated as non-significant (n.s.), * *p* < 0.05, ** *p* < 0.01, and *** *p* < 0.001.

A concise summary of functional and patient-reported outcomes following TKA is provided in Figure 2a–e. Key findings and comparisons of functional tests are provided in Table 1. Both balance measured by the Tinetti score and mobility measured by the TUG test showed a transient, non-significant decline immediately after surgery but recovered to baseline by 6 weeks and improved significantly beyond preoperative levels by 3 and 12 months (Tinetti 15.9 points at baseline vs. 23.9 points after 12 months, *p* < 0.001; TUG at baseline 14.8 s vs. 11.3 s after 12 months, *p* = 0.005 at 12 months). Clinical scoring and assessment measured by KSSp rose steadily from 34.6 points preoperatively to 77.2 points at one year (*p* < 0.001), and the functional component KSSf rebounded from an early postoperative decrease (42.1 points at baseline vs. 26.7 points at days 4–6 after surgery, *p* < 0.001) to exceed baseline by 6 weeks and continue improving to 71.4 points at 12 months (*p* < 0.001). KOOS mirrored these gains, increasing from 38.6% preoperatively to 61.9% at 12 months (*p* < 0.001).

### 3.3. Correlation of Functional Assessments and Patient-Reported Outcomes

There was no significant correlation at any timepoint between HOOS and balance and functional assessment measured by the Tinetti score and the TUG test (Supplementary Table S1). Pearson correlation coefficient showed a moderate correlation between HOOS and HHS at 3 months (r = 0.589, *p* = 0.004) and at 12 months (r = 0.651, *p* < 0.001). Notably, correlation between Tinetti and TUG in the THA group was moderate preoperatively (r = −0.434, *p* = 0.43) and at days 4–6 after surgery (r = −0.426, *p* = 0.48). Furthermore, there was a high correlation between TUG and Tinetti at week 6 (r = −0.778, *p* < 0.001), correlation at 3 months (r = −0.879, *p* < 0.001), and at 12 months (r = −0.863, *p* < 0.001). A comprehensive overview of correlations of the THA is provided in Supplementary Table S1.

In the TKA group, KOOS correlated significantly—but generally at a moderate level—with all other outcome measures, including Tinetti, TUG, and both Knee Society Score subscales, except the KSSp subscale where a high correlation for KOOS was observed at 12 months (r = 0.708, *p* < 0.001). Balance and mobility, Tinetti vs. TUG, were highly correlated preoperatively (r = −0.759, *p* < 0.001), diminished to moderate correlations immediately postoperative (r = −0.569, *p* = 0.007) and at 6 weeks (r = −0.631, *p* = 0.002), then returned to high correlations by 3 months (r = −0.736, *p* < 0.001) and 12 months (r= −0.746, *p* < 0.001). Notably, the KSSf subscale exhibited the highest agreement with Tinetti at 6 weeks (r = 0.822, *p* < 0.001), 3 months (r = 0.923, *p* < 0.001), and 12 months (r = 0.891, *p* < 0.001). An overview of correlations of the TKA is provided in Supplementary Table S2.

### 3.4. Comparative Analysis of Outcomes Following Total Hip and Total Knee Arthroplasty

**Figure 3:**
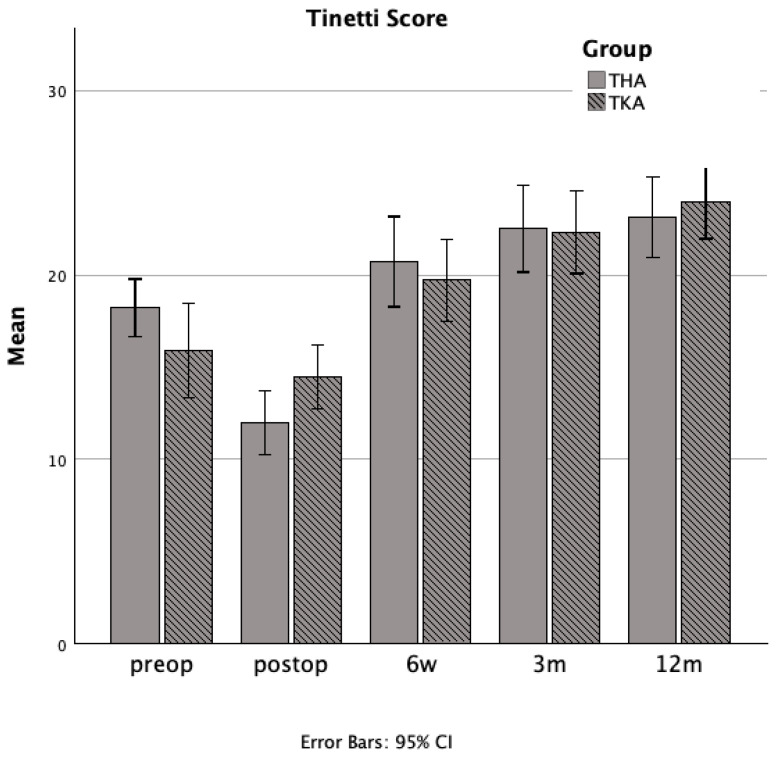
Bar charts showing mean balance performance, as measured by the Tinetti Performance-Oriented Mobility Assessment (POMA), for the total hip arthroplasty group (solid gray bars) and the total knee arthroplasty group (hatched gray bars) at each evaluation time point (preoperative—preop, postoperative days 4–6—postop, six weeks—6 w, three months—3 m, and twelve months—12 m).

**Figure 4:**
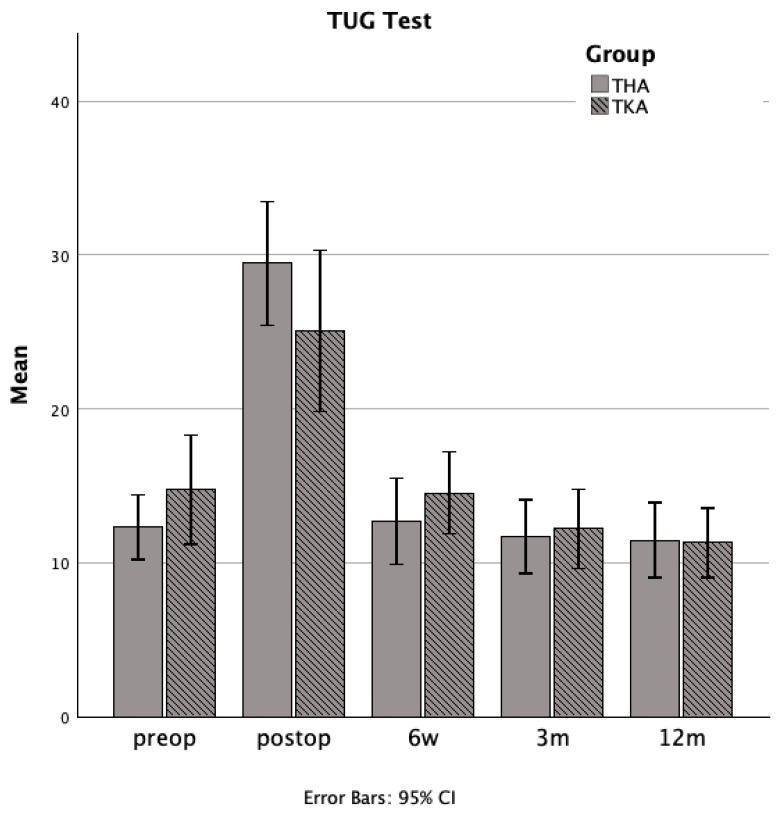
Bar charts showing mean mobility assessments, as measured by the Timed Up and Go (TUG) test, for the total hip arthroplasty group (solid gray bars) and the total knee arthroplasty group (hatched gray bars) at each evaluation time point (preoperative—preop, postoperative days 4–6—postop, six weeks—6 w, three months—3 m, and twelve months—12 m).

Between-group comparisons revealed no statistically significant differences in balance, as measured by the Tinetti score, between the THA and TKA cohorts at any follow-up interval (Figure 3). Likewise, functional mobility assessed by the TUG test did not differ significantly between the two groups at any observational timepoint (Figure 4). Statistical analyses of functional assessments across individual time points are presented in Table 1.

## 4. Discussion

THA and TKA aim to restore function, alleviate pain, enhance mobility, and improve quality of life [2,35,36,37,38]. Sufficient gait and functional balance are important to prevent falling events after joint replacement surgery and by that avoid major complications, especially in an elderly population [39,40]. In this study, both cohorts exhibited a significant decline in balance and mobility immediately after surgery, with recovery to baseline levels by 6 weeks and further improvement at 3 and 12 months postoperatively (*p* < 0.001). PROMs demonstrated earlier improvements, which were sustained over time. While objective measures of balance and mobility showed minimal correlation with PROMs, they were strongly correlated with each other from 6 weeks post-surgery onward.

The Tinetti score represents a sufficient tool to track mobility and risk of falling [21]. However, it is generally not implemented in orthopedic routine. In this study, the time course of balance and risk of falling before and after THA and TKA was shown. As expected both in the THA and TKA group, a decline in the Tinetti score at the immediate postoperative measurement assessed between days 4 and 6 after surgery was detected. The mean score declined to 12.0 points in the THA and 14.4 points in the TKA, respectively. Laurent et al. recently described Tinetti performance in late onset Parkinson’s disease [27]. Patients achieved comparable Tinetti levels between 13 and 14 points when administered to nursing homes due to their leading disease. Furthermore, the Tinetti score was described to be sufficient in monitoring rehabilitation success in stroke patients [41]. At the time of discharge, these patients achieved mean Tinetti scores of 16.2 points. Compared to findings of this study, surgeons should expect a major risk of falling within the first days after THA or TKA, where the performance of postoperative arthroplasty patients might not exceed levels of highly impaired populations with severe diseases [21,42,43]. In time, Tinetti levels raised back to baseline at 6 weeks and exceeded it at 3 months and 12 months, showing the successful impact of joint replacement surgery in both cohorts.

The TUG represents a suitable tool frequently used to monitor the rehabilitation process in many different fields including arthroplasty [18,20,44]. Guttenberg et al. recently aimed to describe a potential cutoff value of preoperative TUG for ambulatory THA, which could not reliably be detected [19]. However, TUG in their study was useful for detected patients with a longer hospital stay. Baseline TUG in our study was comparable to the previously mentioned study. The course of TUG levels in both cohorts showed the poorest level at days 4–6 after surgery as expected. In the THA group, TUG results returned back to baseline at 6 weeks without significantly improving compared to baseline. In the TKA cohort, TUG significantly improved at 3 and 12 months. These results reflect the parallel recovery trajectories in balance and mobility.

Monitoring relevant daily activities of patients is highly relevant in modern arthroplasty research [33,45]. By implementing PROMs, the focus of outcome research became more patient oriented which is equally important in clinical outcome [46,47]. Poitras et al. aimed to assess multiple factors that potentially influence the outcome after arthroplasty. The authors concluded that PROMs may serve as a significant predictor of postoperative outcomes following arthroplasty, underscoring the need for further research to elucidate their prognostic value and optimize their integration into clinical practice [48]. In our study, both HOOS and KOOS exhibited no statistically significant change in the immediate postoperative period, followed by a marked and significant increase at 6 weeks and continued incremental improvements at 3 and 12 months compared with baseline. This temporal pattern of patient-reported outcome gains aligns closely with trajectories reported in previous arthroplasty outcome studies [31,49]. The HHS and KSS—which incorporate patient-reported components of pain and function—demonstrated moderate correlations with their respective PROMs, but predominantly during the later follow-up intervals. In contrast, longitudinal analysis of PROM trajectories versus objective measures of balance and mobility revealed no consistent associations across the entire postoperative recovery period. Balance and mobility results from the THA cohort showed no significant correlation with HOOS throughout the observational period. A comparison of the longitudinal outcome trajectories (Figure 1a–d) reveals a clear dissociation between HOOS and objective performance measures. Whereas HOOS scores begin to improve as early as 6 weeks postoperatively, both the Tinetti score and the TUG decline in the immediate postoperative phase before demonstrating recovery at later follow-up intervals. Similarly, KOOS scores exhibited an upward trend immediately after surgery that reached statistical significance at subsequent follow-up intervals and continued to improve over time. Although KOOS demonstrated moderate correlations with objective measures of mobility and balance, the strength of these associations fell below thresholds typically regarded as clinically meaningful. In both groups, the temporal dissociation indicates that early increases in patient satisfaction and perceived well-being do not reflect true functional capacity or reduced fall risk. Clinicians should therefore counsel patients that, despite early subjective improvements, their balance and mobility remain impaired and fall risk remains elevated, during the initial recovery phase.

A comparison of the THA and TKA cohorts revealed no significant differences in balance or functional outcomes. Certain correlations between PROMs and objective clinical measures were weaker in the THA group compared to the TKA group. This may be attributed to differing recovery trajectories between the two procedures. Patients undergoing THA often experience faster pain relief, yet may initially demonstrate slower improvements in mobility and functional performance during the early recovery phase. Patient satisfaction and function, however, align in the later observational period, which matches the findings in the current literature [47].

Several limitations of this study have to be considered. Selection bias cannot be excluded: participation was voluntary and required ongoing attendance at follow-up visits, potentially favoring patients with higher motivation for postoperative recovery. Nevertheless, the demographic characteristics of both cohorts were comparable and reflective of the typical THA and TKA populations treated at our institution. Furthermore, fall event tracking was not performed, which could have enhanced the clinical relevance and interpretability of the balance assessment findings. Although standardized training and uniform protocols were implemented prior to data collection to minimize inter-observer variability, assessments were performed by multiple examiners, which may have introduced measurement bias. Additionally, this survey was designed as a single-center study, which may introduce bias and limit the external validity of the findings. The absence of a healthy control group limits the ability to directly compare postoperative functional outcomes to normative values. As a result, comparisons were made with data reported in the existing literature. Future studies should consider including a healthy control group to enable more robust benchmarking of recovery trajectories and functional performance. A further limitation of this study is the lack of detailed clinical data such as BMI, comorbidities, ASA classification, frailty scores, and physical activity levels, which may influence functional outcomes and limit the ability to control for potential confounding factors. This study exclusively used HOOS and KOOS as PROMs measurements, without inclusion of more recently discussed instruments such as PROMIS or EQ-5D. This may have offered improved responsiveness and broader applicability in some clinical contexts.

## 5. Conclusions

Postoperative Tinetti balance scores declined significantly in the immediate postoperative period for both THA and TKA cohorts, indicating an elevated risk of falls. Beginning at 6 weeks and continuing through 12 months, balance performance improved steadily and ultimately, exceeded preoperative levels. Longitudinal comparisons between objective balance and functional measures and PROMs demonstrated no clinically meaningful correlations, suggesting that reductions in pain and improvements in perceived well-being occur earlier than measurable gains in balance and mobility. This temporal disconnect may lead patients to overestimate their functional capacity and underestimate residual balance impairments during the early recovery phase.

## Figures and Tables

**Table 1:** Mean values and standard deviations (SD) for key findings from functional performance tests. Within the total hip arthroplasty (THA) and total knee arthroplasty (TKA) columns, results are presented longitudinally relative to the preoperative baseline values for the Tinetti and Timed Up and Go (TUG) tests. These comparisons highlight an immediate postoperative decline in functional performance at days 4–6, followed by progressive improvement at 6 weeks, 3 months, and 12 months. The “THA vs. TKA” column compares mean values between the two groups at each time point, showing no statistically significant differences. Statistical significance is indicated as follows: not significant (n.s.), * *p* < 0.05, ** *p* < 0.01, *** *p* < 0.001.

		THA			TKA			THA vs. TKA
		Mean	(SD)	*p*-Value	Mean	SD	*p*-Value	*p*-Value
Tinetti	preop	18.23	(3.52)		15.90	(5.66)		n.s.
	postop	12.00	(3.94)	***	14.48	(3.78)	n.s.	n.s.
	6 weeks	20.73	(5.52)	n.s.	19.71	(4.87)	**	n.s.
	3 months	22.50	(5.29)	***	22.33	(4.94)	***	n.s.
	12 months	23.14	(4.89)	***	23.95	(4.34)	***	n.s.
TUG	preop	12.31	(4.70)		14.77	(7.81)		n.s.
	postop	29.46	(9.08)	***	25.07	(11.45)	***	n.s.
	6 weeks	12.71	(6.34)	n.s.	14.53	(5.83)	n.s.	n.s.
	3 months	11.70	(5.42)	n.s.	12.21	(5.65)	*	n.s.
	12 months	11.47	(5.50)	n.s.	11.30	(4.97)	**	n.s.

## Data Availability

The data supporting the findings of this study are presented within the manuscript. Additional details are available from the corresponding author upon reasonable request, subject to institutional and ethical guidelines.

## Supplementary Materials

The following supporting information can be downloaded at https://www.mdpi.com/article/10.3390/jcm14124135/s1, Table S1: Pearson correlation coefficients between patient-reported outcomes (PROMs) and functional performance measures in the total hip arthroplasty (THA) cohort. PROMs include the Hip disability and Osteoarthritis Outcome Score (HOOS) and the Harris Hip Score (HHS), while functional performance measures comprise the Tinetti Performance-Oriented Mobility Assessment (POMA) and the Timed Up and Go (TUG) test. Assessments were conducted preoperatively (pre), on postoperative days 4–6 (post), at six weeks (6w), three months (3m), and twelve months (12m) after surgery. Statistical significance is indicated by * for p < 0.05 and ** for p < 0.001.; Table S2: Pearson correlation coefficients between patient-reported outcome measures (PROMs) and objective functional performance assessments in the total knee arthroplasty (TKA) cohort. PROMs comprised the Knee Injury and Osteoarthritis Outcome Score (KOOS) and the Knee Society Score (with separate pain (KSSp) and function (KSSf) subscales), whereas functional performance was evaluated using the Tinetti Performance-Oriented Mobility Assessment (POMA) and the Timed Up and Go (TUG) test. Assessments were conducted preoperatively (pre), on postoperative days 4–6 (post), at six weeks (6w), three months (3m), and twelve months (12m) after surgery. Statistical significance is indicated by * for p < 0.05 and ** for p < 0.01.

## Abbreviations

The following abbreviations are used in this manuscript.

HHS	Harris Hip Score
HOOS	Hip Disability and Osteoarthritis Outcome Score
KOOS	Knee Injury and Osteoarthritis Outcome Score
KSS	Knee Society Score
KSSf	Knee Society Score subscale for function
KSSp	Knee Society Score subscale for pain
n.s.	non-significant
POMA	Tinetti Performance-Oriented Mobility Assessment
PROMs	patient-reported outcome measures
SD	Standard deviation
THA	Total Hip Arthroplasty
TKA	Total Knee Arthroplasty
TUG	Timed Up and Go
