# Supplementary material for: Balance and Mobility in Comparison to Patient-Reported Outcomes—A Longitudinal Evaluation After Total Hip and Knee Arthroplasty
Source: J Clin Med. 2025 Jun 11;14(12):4135. doi: 10.3390/jcm14124135 (PMC12193784; doi:10.3390/jcm14124135)
Supplement: Supplementary file 1 [file jcm-14-04135-s001.zip › jcm-3639911-supplementary.pdf]

Supplementary material for “Balance and Mobility in Comparison to Patient-Reported Outcomes – A Longitudinal Evaluation after Total Hip and Knee Arthroplasty”

Correlations THA																					
		HOOS					Tinetti					TUG					HHS				
		pre	post	6w	3m	12m	pre	post	6w	3m	12m	pre	post	6w	3m	12m	pre	post	6w	3m	12m
HOOS	pre	1					-0.031					-0.146					0.354				
	post		1					0.376					-0.020					0.176			
	6w			1					0.237					-0.163					0.341		
	3m				1					0.269					-0.341					**0.589****	
	12m					1					0.343					-0.350					**0.651****
Tinetti	pre	-0.031					1					-0.434*					0.145				
	post		0.376					1					-0.426*					0.259			
	6w			0.237					1					-**0.778****					**0.764****		
	3m				0.269					1					-**0.879****					**0.732****	
	12m					0.343					1					-**0.863****					**0.714****
TUG	pre	-0.146					-0.434*					1					-0.326				
	post		-0.020					-0.426*					1					-0.261			
	6w			-0.163					-**0.778****					1					-**0.621****		
	3m				-0.341					-**0.879****					1					-**0.830****	
	12m					-0.350					-**0.863****					1					-**0.781****
HHS	pre	0.354					0.145					-0.326					1				
	post		0.176					0.259					-0.261					1			
	6w			0.341					**0.764****					-**0.621****					1		
	3m				**0.589****					**0.732****					-**0.830****					1	
	12m					**0.651****					**0.714****					-**0.781****					1

Supplementary Table S1: Pearson correlation coefficients between patient-reported outcomes (PROMs) and functional performance measures in the total hip arthroplasty (THA) cohort. PROMs include the Hip disability and Osteoarthritis Outcome Score (HOOS) and the Harris Hip Score (HHS), while functional performance measures comprise the Tinetti Performance-Oriented Mobility Assessment (POMA) and the Timed Up and Go (TUG) test. Assessments were conducted preoperatively (pre), on postoperative days 4–6 (post), at six

weeks (6w), three months (3m), and twelve months (12m) after surgery. Statistical significance is indicated by * for $p < 0.05$ and ** for $p < 0.001$.

Correlations TKA																										
		KOOS					Tinetti					TUG					KSSp					KSSf				
		pre	post	6w	3m	12m	pre	post	6w	3m	12m	pre	post	6w	3m	12m	pre	post	6w	3m	12m	pre	post	6w	3m	12m
KOOS	pre	1					0.508*					**-0.592****					0.485*					0.470*				
	post		1					0.438*					**-0.625****					0.156					0.170			
	6w			1					**0.598****					**-0.644****					**0.671****					**0.586****		
	3m				1					0.521*					**-0.600****					**0.640****					**0.603****	
	12m					1					0.469*					**-0.597****					**0.708****					**0.552****
Tinetti	pre	0.508*					1					**-0.759****					0.299					0.357				
	post		0.438*					1					**-0.569****					0.239					0.333			
	6w			**0.598****					1					**-0.631****					0.494*					**0.822****		
	3m				0.521*					1					**-0.736****					**0.568****					**0.923****	
	12m					0.469*					1					**-0.746****					0.416					**0.891****
TUG	pre	**-0.592****					**-0.759****					1					-0.428					-0.391				
	post		**-0.625****					**-0.569****					1					-0.467*					-0.303			
	6w			**-0.644****					**-0.631****					1					-0.375					**-0.680****		

	3m				**-0.600****					**-0.736****					1					-0.426					**-0.748****	
	12m					**-0.597****					**-0.746****					1					-0.408					**-0.732****
KSSp	pre	0.485*					0.299					-0.428					1					0.083				
	post		0.156					0.239					-0.467*					1					0.305			
	6w			**0.671****					0.494*					-0.375					1					**0.555****		
	3m				**0.640****					**0.568****					-0.426					1					**0.715****	
	12m					**0.708****					0.416					-0.408					1					**0.645****
KSSf	pre	0.470*					0.357					-0.391					0.083					1				
	post		0.170					0.333					-0.303					0.305					1			
	6w			**0.586****					**0.822****					**-0.680****					**0.555****					1		
	3m				**0.603****					**0.923****					**-0.748****					**0.715****					1	
	12m					**0.552****					**0.891****					**-0.732****					**0.645****					1

Supplementary Table S2: Pearson correlation coefficients between patient-reported outcome measures (PROMs) and objective functional performance assessments in the total knee arthroplasty (TKA) cohort. PROMs comprised the Knee Injury and Osteoarthritis Outcome Score (KOOS) and the Knee Society Score (with separate pain (KSSp) and function (KSSf) subscales), whereas functional performance was evaluated using the Tinetti Performance-Oriented Mobility Assessment (POMA) and the Timed Up and Go (TUG) test. Assessments were conducted preoperatively (pre), on postoperative days 4–6 (post), at six weeks (6w), three months (3m), and twelve months (12m) after surgery. Statistical significance is indicated by * for $p < 0.05$ and ** for $p < 0.01$.
